# Long-term trajectories and cumulative exposure of the triglyceride–glucose–frailty index in relation to hip fracture risk: evidence from a large-scale population-based cohort

**DOI:** 10.1007/s11657-026-01683-z

**Published:** 2026-05-08

**Authors:** Han Su, Chengfeng Fu, Pingping Wang, Yingying Zhang

**Affiliations:** 1 Department of Orthopedics, The Central Hospital Affiliated to Jiangnan University, Wuxi, Jiangsu China; 2 Respiratory and Critical Care Medicine, The Second People’s Hospital of Banan District, Chongqing, China; 3 Department of Clinical Laboratory, Taizhou Second People’s Hospital Affiliated to Yangzhou University, Taizhou, Jiangsu China; 4 Medical Laboratory Center, Affiliated Wuxi Fifth Hospital of Jiangnan University, Wuxi, Jiangsu China; 5 Department of Clinical Laboratory, The Second Affiliated Hospital of Chongqing Medical University, Chongqing, China

**Keywords:** Triglyceride–glucose, Frailty, Population-based cohort, Metabolic syndrome, Hip fractures

## Abstract

***Summary*:**

Long-term trajectories and cumulative burden of the triglyceride–glucose–frailty index (TyGFI) exhibited nonlinear associations with hip fracture risk. Persistent metabolic–frailty imbalance markedly increased fracture susceptibility, identifying TyGFI as a potential risk factor for early detection and prevention of hip fracture.

**Background:**

Hip fracture is a major cause of disability and mortality in older adults, yet traditional risk factors explain only part of its variability. Metabolic dysfunction and frailty may jointly contribute to skeletal fragility. The triglyceride–glucose–frailty index (TyGFI) integrates these domains, but its long-term association with hip fracture risk remains unclear.

**Methods:**

A total of 6130 adults aged ≥ 45 years from the China Health and Retirement Longitudinal Study were included. TyGFI was calculated as the product of the triglyceride–glucose index and frailty index. Participants were grouped into longitudinal trajectories using *k*-means clustering, and cumulative exposure was estimated as the area under the curve between 2012 and 2015. Cox regression and spline analyses evaluated associations with hip fracture risk.

**Results:**

Three distinct TyGFI trajectories were identified—low–stable, moderate–increasing, and high–increasing. Compared with the low–stable group, participants in the moderate– and high–increasing groups had higher hip fracture risks (HR = 1.97 and 3.79, both *P* < 0.001). Cumulative TyGFI showed a nonlinear association with fracture risk, with a potential threshold around 4.5. Incorporating cumTyGFI significantly improved predictive performance (*C*-statistic = 0.7340; NRI = 0.4330; IDI = 0.0097; all *P* < 0.001). Results were robust across subgroups and multiple sensitivity analyses.

**Conclusions:**

Long-term trajectories and cumulative exposure of TyGFI were independently associated with hip fracture risk, suggesting that persistent metabolic–frailty imbalance contributes to skeletal fragility. TyGFI may serve as a practical integrative marker for early identification and prevention of high-risk individuals.

**Supplementary Information:**

The online version contains supplementary material available at 10.1007/s11657-026-01683-z.

## Introduction

Hip fracture is the most devastating form of osteoporotic fracture in later life and imposes a substantial and escalating clinical and socioeconomic burden worldwide. The Global Burden of Disease Study 2019 reported that the age-standardized incidence of hip fracture has increased by approximately 0.4% annually, and the global case number is projected to nearly double from 3.16 million in 2018 to 6.26 million by 2050 [1, 2]. Despite advances in surgical management and post-fracture care, one-year mortality remains high (often exceeding 20–30%) [3]. China exemplifies this global trend, where rapid population aging has led to a steep rise in hip fracture incidence, underscoring the urgent need for early identification and prevention among high-risk individuals [4].

Traditional risk factors—such as age, sex, bone mineral density (BMD), and prior falls—constitute the cornerstone of fracture risk assessment, yet they account for only part of the variability in fracture susceptibility [5–7]. Notably, a substantial proportion of fragility fractures occur among individuals whose BMD *T*-scores remain above the diagnostic threshold for osteoporosis (*T* > − 2.5) [8–10]. This observation suggests that systemic metabolic and physiological vulnerability contributes to skeletal fragility beyond bone mass reduction alone.

Metabolic dysregulation is increasingly recognized as a determinant of skeletal fragility. Insulin resistance, obesity, and metabolic syndrome may impair bone strength through chronic inflammation, oxidative stress, disrupted bone remodeling, and deterioration of bone microarchitecture [11–14]. Consistent with the “diabetic bone paradox,” individuals with type 2 diabetes mellitus (T2DM) may exhibit normal or even higher BMD yet remain at elevated fracture risk. A key mechanistic explanation involves the progressive accumulation of advanced glycation end products (AGEs) within long-lived bone matrix proteins—particularly type I collagen—under chronic hyperglycemia [15]. AGE-mediated nonenzymatic collagen cross-linking and cellular signaling disturbances can impair bone material quality and mineralization, thereby increasing fracture susceptibility despite preserved or even elevated BMD [15]. Together, these findings highlight that metabolic abnormalities may influence fracture risk through pathways beyond bone mass reduction [16, 17].

Beyond metabolic perturbations, frailty reflects multisystem deficit accumulation and reduced physiological reserve and is a recognized determinant of falls and fractures in older adults. The Frailty Index (FI) operationalizes frailty as a deficit accumulation measure of biological aging and vulnerability, with items constructed based on cohort-available variables. In parallel, the triglyceride–glucose (TyG) index—derived from fasting triglycerides and glucose—is a simple surrogate marker of insulin resistance and has been associated with fragility fracture risk [18]. However, most existing investigations rely on single time-point TyG measurements, limiting the ability to characterize long-term or cumulative metabolic burden and motivating studies incorporating longitudinal patterns and cumulative exposure [19].

Recently, the triglyceride–glucose–frailty index (TyGFI) has been proposed as an integrated indicator combining the TyG index and FI to capture joint metabolic–frailty burden beyond either component alone [20]. In this framework, TyGFI is estimated by integrating insulin resistance–related metabolic burden (TyG) with deficit accumulation frailty (FI), thereby reflecting the combined impact of metabolic dysfunction and physiological vulnerability relevant to fracture susceptibility. Given shared pathways linking metabolic dysfunction and frailty to skeletal vulnerability—including chronic inflammation, sarcopenia, and endocrine dysregulation—TyGFI may provide a more comprehensive assessment of long-term fracture risk [21, 22]. Supporting this integrative rationale, a CHARLS-based study showed that individuals with both frailty and higher TyG—and those with higher cumulative levels of both—had the highest cardiometabolic risk [23]. Nevertheless, evidence specific to TyGFI remains limited and has primarily focused on cardiovascular endpoints; whether TyGFI predicts osteoporotic fractures has not been systematically evaluated [20]. Accordingly, longitudinal evidence assessing TyGFI patterns and cumulative exposure in relation to incident hip fracture risk is still lacking.

To address this gap, we conducted a nationwide cohort study using the CHARLS, a representative sample of middle-aged and older Chinese adults. TyGFI control levels were characterized using *k*-means clustering, and cumulative exposure was quantified using an area-under-the-curve approach (hereafter referred to as cumTyGFI). We then examined associations of TyGFI control levels and cumTyGFI with incident hip fracture risk. We hypothesized that persistently elevated metabolic–frailty burden, reflected by adverse TyGFI control levels and higher cumTyGFI, would be associated with increased hip fracture risk independent of demographic, metabolic, and lifestyle factors.

## Methods

### Study design and population

This study utilized data from the CHARLS, a nationally representative, prospective cohort investigating the health, socioeconomic, and demographic characteristics of Chinese adults aged ≥ 45 years [24]. CHARLS recruited participants through multistage probability sampling across 28 provinces in China. Five survey waves have been completed to date, beginning with the baseline survey in 2011 (Wave 1) and followed by subsequent follow-ups in 2013 (Wave 2), 2015 (Wave 3), 2018 (Wave 4), and 2020 (Wave 5) [24]. Fasting blood samples and other biomarker data were collected during Waves 1 and 3. Therefore, fasting biomarker data were available only for respondents who consented to and successfully completed the biomarker collection in these waves. The CHARLS protocol was approved by the Institutional Review Board of Peking University (IRB00001052-11015), and written informed consent was obtained from all participants prior to enrollment.

**Fig. 1:**
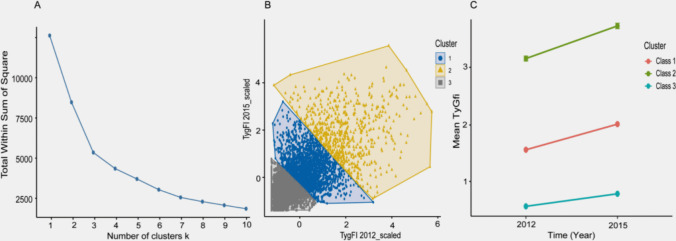
Identification of TyGFI control levels using *k*-means clustering. **A** Elbow plot of the within-cluster sum of squares (WCSS) across different numbers of clusters (*K*); the inflection point at *K* = 3 indicated the optimal number of clusters. **B** Scatter plot of participants according to standardized TyGFI values in 2012 and 2015, colored by k-means cluster assignment. **C** Mean TyGFI values in 2012 and 2015 within each control level (lines connect the two time points): Class 1, moderate–increasing; Class 2, high–increasing; and Class 3, low–stable

At baseline, 17,705 participants were enrolled in Wave 1. Participants were excluded if they lacked fasting blood samples in either 2011–2012 (*n* = 5858) or 2015 (*n* = 4267), had missing data required to calculate the TyG index or frailty index (*n* = 1267), reported a history of hip fracture before or in 2015 (*n* = 127), or were younger than 45 years (*n* = 56). After applying these criteria, 6130 eligible participants were included in the final analysis. The overall participant selection flow is shown in Fig. 1.

### Data collection and indicator definitions

Fasting venous blood samples were obtained after an overnight fast of at least 8 h. Fasting blood glucose (FBG) and triglyceride (TG) levels were measured using an enzymatic colorimetric method on an automated chemistry analyzer (Hitachi 7180, Tokyo, Japan). The TyG, a surrogate marker of insulin resistance, was calculated as: TyG = ln [(TG (mg/dL) × FBG (mg/dL))/2]. The FI was constructed using the deficit accumulation model, incorporating 30 health deficits across domains including chronic diseases, physical function, depressive symptoms, and cognitive performance (Supplementary Table 1). Each variable was coded as binary (deficit = 1 and no deficit = 0) or continuous on a 0–1 scale, and FI was computed as the ratio of deficits to total items [25, 26]. TyGFI was derived using a multiplicative model to capture the joint metabolic–frailty burden: TyGFI = TyG × FI. cumTyGFI was estimated as the area under the curve (AUC) for TyGFI between 2012 and 2015 using the trapezoidal rule: cumTyGFI = [(TyGFI_2012 + TyGFI_2015)/2] × (2015–2012). The cumTyGFI was used as a descriptive variable name for this cumulative exposure metric, consistent with commonly used AUC-based measures (e.g., cumTyG) in longitudinal studies [27].

To characterize between-wave changes in metabolic–frailty burden, TyGFI control levels (cluster-derived groups) were identified using an unsupervised *k*-means clustering approach based on standardized TyGFI values measured in 2012 and 2015. As shown in the elbow plot (Fig. 1A), the marginal reduction in within-cluster sum of squares (WCSS) diminished substantially beyond *K *= 3, indicating an elbow point where additional clusters provided only minimal improvement; therefore, *K* = 3 was selected as the optimal solution. Participants were assigned to the nearest centroid (cluster mean) in the standardized TyGFI feature space (Fig. 1B); thus, no prespecified cutoff values were used to define TyGFI control levels [28]. The resulting control levels were labeled according to their mean TyGFI level and direction of change between 2012 and 2015 (low–stable, moderate–increasing, and high–increasing). The final sizes of each control level are reported in the “ Results” section and shown in Fig. 1. For clarity, “TyGFI control levels” denote *k*-means–derived level-change profiles rather than prespecified clinical control targets or cutoff-based categories.

### Outcome determination

The primary outcome was incident hip fracture. Participants who reported a history of hip fracture before or during the 2015 assessment were excluded at baseline. Incident hip fractures were ascertained during the 2018 and 2020 follow-up waves based on participants’ responses to the standardized question, “Since our last interview, have you fractured your hip?” Because exact event dates are not recorded in CHARLS, fracture time was approximated as the midpoint between the last fracture-free visit and the first fracture-reported visit, an established approach to reduce event-time misclassification in longitudinal cohort studies [29]. Follow-up (time zero) began at the 2015 assessment (Wave 3), when TyGFI control levels and cumTyGFI were defined. Participants who did not report an incident hip fracture were censored at their last completed follow-up interview [29]. 

### Covariates

All covariates were derived from the CHARLS 2011 baseline survey (Wave 1) and covered a broad range of sociodemographic, behavioral, and health-related characteristics. Sociodemographic variables included age, sex (male or female), education level (less than lower secondary or upper secondary and above), residence (rural or urban), and marital status, which was categorized as married or partnered versus unmarried (including separated, divorced, widowed, or never married). Health behavior variables comprised current smoking (yes or no), alcohol consumption (never, less than once per month, and more than once per month), and physical activity (light and moderate-to-vigorous). Comorbidity was defined as the number of nine self-reported chronic conditions—hypertension, diabetes, dyslipidemia, chronic kidney disease, heart disease, stroke, chronic lung disease, arthritis, and cancer—and was grouped as 0, 1, or ≥2. Although arthritis was included in the comorbidity count, it was additionally adjusted for because of its strong link to impaired function and fall risk.

### Statistical analysis

Baseline characteristics were summarized as mean ± standard deviation (SD) or median (interquartile range (IQR)) for continuous variables, and as counts (percentages) for categorical variables. Differences across TyGFI control levels were assessed using one-way analysis of variance (ANOVA) for normally distributed variables, the Kruskal–Wallis test for skewed variables, and the chi-square test for categorical variables.

Missing covariate data were handled using multiple imputation by chained equations (MICE), with five imputations. Cox proportional hazards models were applied to examine associations of TyGFI control levels and cumTyGFI with incident hip fracture among participants free of hip fracture at baseline. cumTyGFI was analyzed as a continuous variable and by quartiles (Q1–Q4), and hazard ratios (HRs) with 95% confidence intervals (CIs) were estimated. Three hierarchically adjusted Cox models were constructed. Model 1 adjusted for demographic and socioeconomic factors (age, sex, education, marital status, and residence). Model 2 additionally adjusted for lifestyle behaviors (smoking, alcohol consumption, and physical activity). Model 3 further adjusted for clinical conditions, including comorbidity (0, 1, or ≥2 chronic diseases) and arthritis.

Kaplan–Meier curves and log-rank tests were used to compare cumulative incidence of hip fracture across TyGFI control levels and cumTyGFI quartiles. To explore potential nonlinear associations, restricted cubic spline (RCS) functions were fitted within the Cox models, with knots placed at the 5th, 50th, and 95th percentiles of cumTyGFI. Nonlinearity was assessed using likelihood ratio tests comparing models with and without spline terms. Possible threshold effects were further evaluated using segmented Cox regression, where the optimal turning point of cumTyGFI was determined by likelihood ratio testing between single-line and two-segment models; HRs were estimated separately below and above this data-driven cutoff.

Model discrimination and predictive improvement were evaluated using the concordance index (*C*-statistic), integrated discrimination improvement (IDI), and net reclassification improvement (NRI). The *C*-statistic assessed overall discrimination, while IDI and NRI quantified incremental predictive value after incorporating TyGFI control levels or cumTyGFI. Model performance was further examined by comparing changes in the *C*-statistic and the area under the receiver operating characteristic curve (AUC), along with corresponding IDI and NRI estimates.

To enhance interpretability, SHapley Additive exPlanations (SHAP) analysis was used to decompose model predictions into the marginal contribution of each variable. Global SHAP values quantified the relative importance of predictors for hip fracture risk, and the six most influential variables were visualized using summary bar and beeswarm plots, illustrating both the direction and magnitude of their effects.

Several sensitivity analyses were performed to assess robustness. First, subgroup analyses were stratified by age, sex, residence, education, smoking, alcohol use, body mass index (underweight < 18.5 kg/m^2^, normal weight 18.5–24.9 kg/m^2^, and overweight/obese ≥ 25 kg/m^2^), arthritis, hypertension, and diabetes, with interactions tested using likelihood ratio tests. Second, analyses were repeated using complete-case data to assess the influence of missing values. Third, analyses were repeated after excluding participants with baseline diabetes and dyslipidemia. Finally, an alternative wave-based time scale was applied to verify robustness of event–time estimation.

## Results

### Identification of TyGFI control levels

Using an unsupervised *k*-means clustering approach (based on standardized TyGFI values measured in 2012 and 2015), three TyGFI control levels were identified (*K* = 3): Class 1 (moderate–increasing, *n* = 2137), Class 2 (high–increasing, *n *= 605), and Class 3 (low–stable, *n* = 3,388) (Fig. 1). The WCSS elbow method supported *K* = 3 as the optimal solution. Class 1 (moderate–increasing) comprised 2137 participants (34.9%) with intermediate TyGFI levels that increased from 1.53 ± 0.52 in 2012 to 1.97 ± 0.64 in 2015. Class 2 (high–increasing) included 605 participants (9.9%) with high TyGFI levels that further increased from 3.11 ± 0.71 to 3.66 ± 0.82. Class 3 (low–stable) encompassed 3388 participants (55.3%) with consistently low TyGFI levels (0.55 ± 0.38 to 0.76 ± 0.44). These cluster-derived control levels were used as the exposure in subsequent analyses of incident hip fracture risk.

### Baseline characteristics

Baseline characteristics of the 6130 participants by TyGFI control level are presented in Supplementary Table S2. Significant between-group differences were observed across the three TyGFI control levels (all *P* < 0.001). Participants in the high–increasing control level tended to be older and more often female, had lower educational attainment, and showed higher prevalences of hypertension, diabetes, dyslipidemia, and arthritis, along with higher fasting glucose and triglyceride levels. In contrast, participants in the low–stable control level were younger, more often male, and exhibited more favorable metabolic profiles, lower FI levels, and lower comorbidity burden; the moderate–increasing control level showed intermediate characteristics.

#### Associations of TyGFI control levels and cumulative exposure with hip fracture

**Fig. 2:**
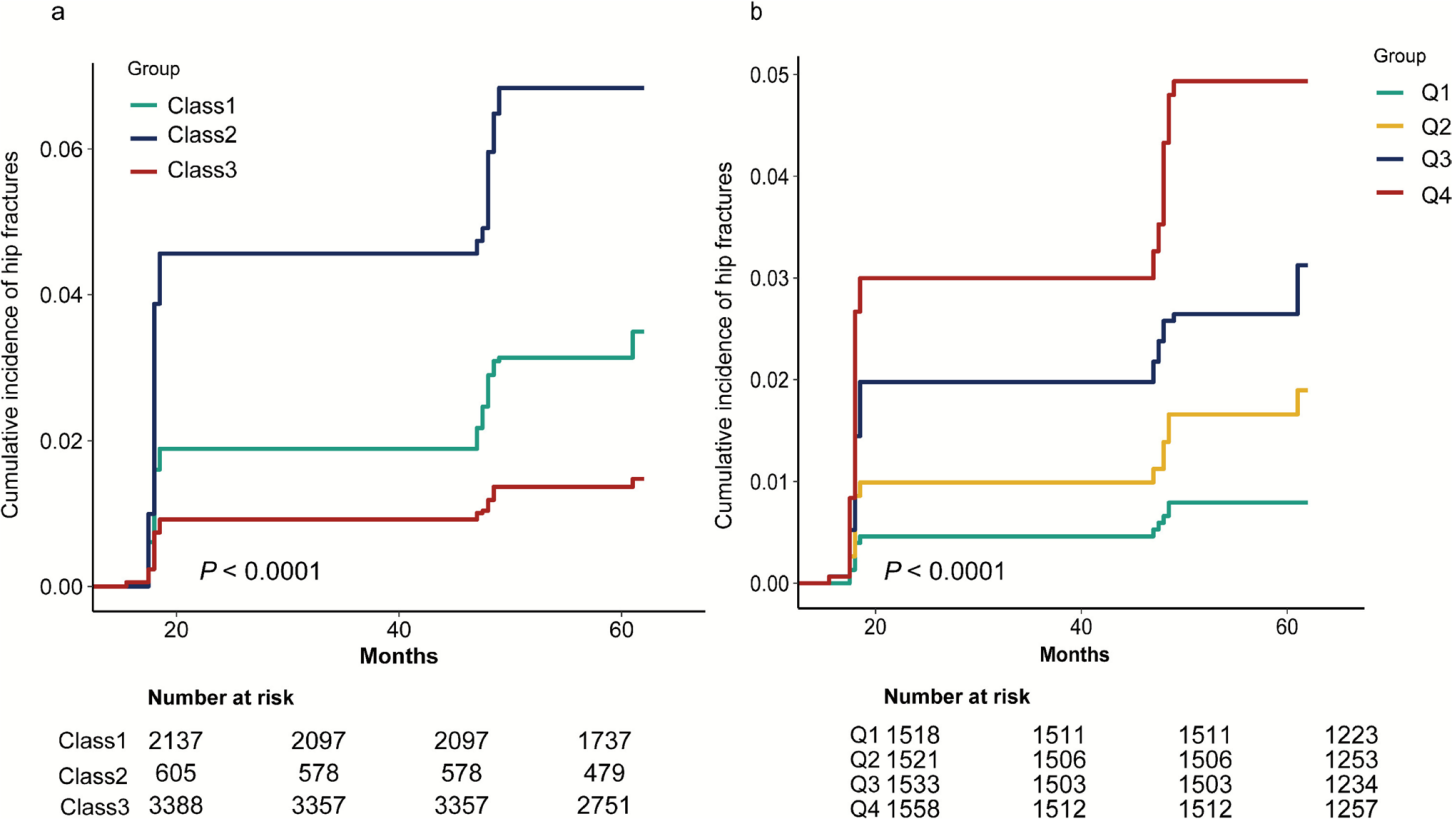
Cumulative incidence of hip fracture by TyGFI control levels and cumulative exposure. (A) Cumulative incidence of hip fracture across TyGFI control levels (Class 1, moderate–increasing; Class 2, high–increasing; and Class 3, low–stable). (B) Cumulative incidence of hip fracture across cumTyGFI quartiles (Q1–Q4). *P* values are from log-rank tests. Follow-up started at the 2015 assessment (Wave 3) and hip fractures were ascertained at the 2018 and 2020 waves (Waves 4 and 5). Because exact fracture dates are not available in CHARLS, event time was approximated as the midpoint between consecutive waves; therefore, curve steps may cluster around the midpoints of each interval

Participants were followed from 2015 (Wave 3) through the 2018 and 2020 follow-up waves (Waves 4 and 5), corresponding to approximately 3- and 5-year follow-up windows. Hip fracture risk differed significantly across TyGFI control levels and cumTyGFI quartiles (log-rank *P* < 0.0001; Fig. 2). Compared with the low–stable control level, the moderate–increasing and high–increasing control levels had approximately twofold (adjusted HR = 1.97, 95% CI 1.29–3.02) and 3.79-fold (adjusted HR = 3.79, 95% CI 2.26–6.53) higher hip fracture risks, respectively (Table 1). Each one-unit increment in cumTyGFI was associated with an 18% increase in hip fracture risk (adjusted HR = 1.18, 95% CI 1.12–1.24; *P* < 0.001). A clear dose–response trend was evident across cumTyGFI quartiles (Q2: HR = 2.10; Q3: HR = 3.19; Q4: HR = 5.46; *P* for trend < 0.001).

**Table 1:** Associations of TyGFI trajectory classes and cumulative TyGFI with risk of incident hip fracture

Variables	Crude model		Model 1^a^		Model 2^b^		Model 3^c^	
	HR (95% CI)	*P* value	HR (95% CI)	*P* value	HR (95% CI)	*P* value	HR (95% CI)	*P* value
**TyGFI control levels**								
**Class 3 (low–stable)**	1 (Ref)		1 (Ref)		1 (Ref)		1 (Ref)	
**Class 1 (moderate–increasing)**	2.35 (1.62 ~ 3.41)	< 0.001	1.92 (1.31 ~ 2.81)	0.001	1.95 (1.33 ~ 2.86)	0.001	1.97 (1.29 ~ 3.02)	0.002
**Class 2 (high–increasing)**	5.00 (3.28 ~ 7.63)	< 0.001	3.62 (2.32 ~ 5.66)	< 0.001	3.69 (2.36 ~ 5.78)	< 0.001	3.79 (2.26 ~ 6.35)	< 0.001
**cumTyGFI continuous**	1.20 (1.15 ~ 1.24)	< 0.001	1.17 (1.12 ~ 1.22)	< 0.001	1.17 (1.12 ~ 1.22)	< 0.001	1.18 (1.12 ~ 1.24)	< 0.001
**cumTyGFI quartile**								
**Q1**	1 (Ref)		1 (Ref)		1 (Ref)		1 (Ref)	
**Q2**	2.17 (1.09 ~ 4.3)	0.026	1.91 (0.96 ~ 3.79)	0.065	1.90 (0.96 ~ 3.78)	0.066	2.10 (1.04 ~ 4.25)	0.039
**Q3**	3.53 (1.86 ~ 6.71)	< 0.001	2.79 (1.46 ~ 5.34)	0.002	2.83 (1.48 ~ 5.43)	0.002	3.19 (1.58 ~ 6.42)	0.001
**Q4**	6.37 (3.46 ~ 11.71)	< 0.001	4.52 (2.41 ~ 8.46)	< 0.001	4.62 (2.46 ~ 8.66)	< 0.001	5.46 (2.69 ~ 11.09)	< 0.001
***P* for trend**		< 0.001		< 0.001		< 0.001		< 0.001

^a^Model 1 was adjusted for age, sex, education level, marital status and residence

^b^Model 2 was further adjusted for smoking status, alcohol consumption and physical activity

^c^Model 3 was further adjusted for comorbidity (0/1/≥ 2) and arthritis

*HR* hazard ratio, *CI* confidence interval, *Ref* reference, *cumTyGFI* cumulative triglyceride-glucose and frailty index

Restricted cubic splines indicated a significant nonlinear association between cumTyGFI and hip fracture risk (overall *P* < 0.001; *P* for nonlinearity = 0.027; Fig. S1). A piecewise Cox model using a turning point at cumTyGFI = 4.5 showed a stronger association below 4.5 (adjusted HR = 1.434, 95% CI 1.106–1.859) and an attenuated association above 4.5 (adjusted HR = 1.075, 95% CI 0.980–1.178), with evidence of a slope change (likelihood ratio test *P* = 0.002) (Table 2).

**Table 2:** Piecewise association between cumulative TyGFI (cumTyGFI) and incident hip fracture risk with a model-derived inflection point at cumTyGFI = 4.5

cumTyGFI (per 1-unit increase)	Crude model		Adjusted model^a^	
	HR (95% CI)	*P* value	HR (95% CI)	*P* value
< 4.5	1.455 (1.177, 1.8)	< 0.001	1.434 (1.106, 1.859)	0.0066
≥ 4.5	1.077 (0.984, 1.179)	0.106	1.075 (0.98, 1.178)	0.1242
Likelihood ratio test (change in slope)		0.015		0.002

^a^Adjusted for age, sex, residential area, education level, marital status, smoking, alcohol consumption, comorbidity burden, and arthritis.

The likelihood ratio test compared a linear Cox model with a two-segment piecewise Cox model (knot at cumTyGFI = 4.5).* P* values reflect evidence of a change in slope at the knot; 4.5 was treated as a model-derived inflection point rather than a clinical cutoff

### Subgroup analyses

**Fig. 3:**
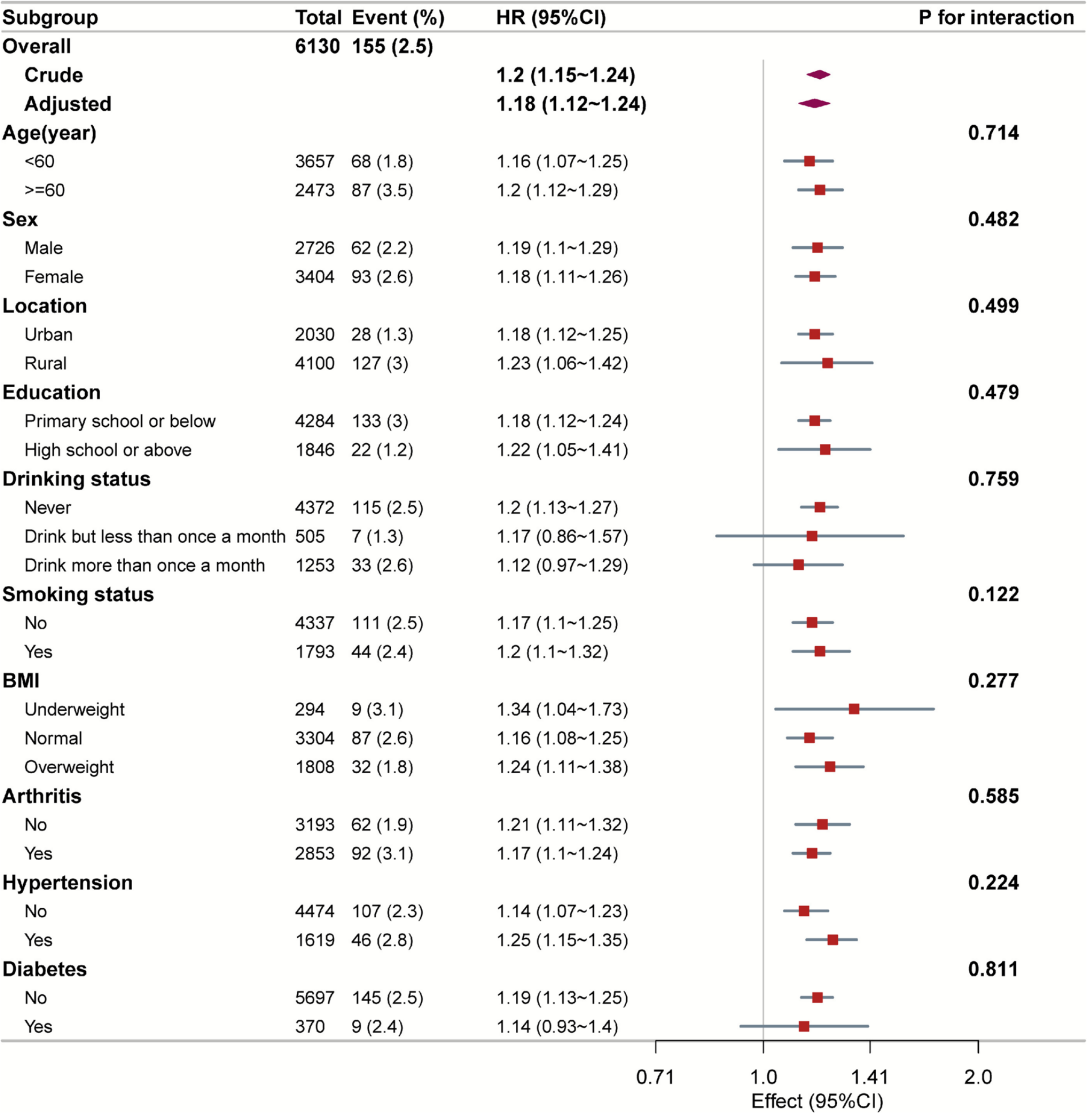
Subgroup analyses of the association between cumulative TyGFI and the risk of incident hip fracture. Analyses were based on Model 3, with the stratification variable itself not included in the adjustment

In multivariable stratified analyses, the associations of both TyGFI control levels and cumTyGFI with hip fracture risk remained broadly consistent across subgroups defined by age, sex, residence, education, smoking, alcohol use, BMI, arthritis, hypertension, and diabetes (Fig. 3). Compared with the low–stable control level, both the moderate–increasing and high–increasing control levels were generally associated with higher hip fracture risk across prespecified subgroups. No statistically significant effect modification was observed (all *P* for interaction > 0.10; Supplementary Table S3).

### Incremental predictive value of TyGFI and related indicators

Incorporation of cumTyGFI into the baseline model improved discrimination for hip fracture (Table 3 and Fig. S3). The *C*-statistic increased from 0.6856 to 0.7340 (*P* < 0.001), accompanied by significant enhancements in risk reclassification (NRI = 0.4330, *P* < 0.001) and discrimination (IDI = 0.0097, *P* < 0.001). Models including TyG, fasting glucose, or triglycerides alone yielded minimal improvements (*P* > 0.05). When evaluated at baseline, adding FI improved discrimination (*C*-statistic = 0.7198), while baseline TyGFI yielded a comparable improvement (*C*-statistic = 0.7174).

**Table 3:** Incremental predictive value of TyG and cumTyGFI beyond the baseline risk model for incident hip fracture (*C*-statistic, NRI, and IDI)

Model	*C*-statistic (95CI%)	*P* value	NRI (95CI%)	*P* value	IDI (95CI%)	*P* value
Baseline risk model	0.6856 (0.6459–0.7252)		Reference		Reference	
+ cumTyGFI	0.7340 (0.6958–0.7723)	< 0.001	0.4330 (0.2746–0.5915)	< 0.001	0.0097 (0.0047–0.0148)	0.00017
+ TyG	0.6928 (0.6531–0.7324)	0.102	0.1045 (−0.0532–0.2622)	0.19402	0.0009 (0–0.0018)	0.0567
+ FI	0.7198 (0.6805–0.7591)	0.004	0.3128 (0.1537–0.4719)	0.00012	0.0092 (0.0041–0.01)	< 0.001
+ Glucose	0.6932 (0.6539–0.7324)	0.134	0.1569 (0.0093–0.3045)	0.0372	0.0011 (0.0001–0.002)	0.0376
+ Triglycerides	0.6880 (0.6482–0.7278)	0.343	0.1532 (−0.0031–0.3094)	0.0547	0.0002 (−0.0003–0.0006)	0.41031
+ TyGFI_2012_	0.7174 (0.6782–0.7565)	0.004	0.3024 (0.1433–0.4615)	< 0.001	0.0076 (0.0031–0.0121)	0.00102

Baseline risk model included age, sex, education level, marital status, residence, smoking status, drinking status, comorbidity, and arthritis. *C*-statistic values are presented with 95% confidence intervals. *NRI* indicates net reclassification improvement, *IDI* indicates integrated discrimination improvement

### Variable importance analysis based on SHAP values

SHAP analysis identified cumTyGFI as the most influential predictor of hip fracture, followed by age and residence (Fig. S4). In contrast, education, smoking, and alcohol consumption contributed minimally. Higher cumTyGFI and older age were strongly associated with increased hip fracture risk, whereas urban residence and being married were associated with lower risk. These findings highlight the central role of cumulative metabolic–frailty burden in skeletal fragility beyond traditional demographic and lifestyle factors.

### Sensitivity analyses

All sensitivity analyses supported the robustness of the findings. Complete-case analyses, after excluding participants with missing covariates, yielded results consistent with the main analysis, with higher-risk TyGFI control levels and higher cumTyGFI remaining significantly associated with increased hip fracture risk (Supplementary Table S4). In analyses restricted to participants free of both diabetes and dyslipidemia at baseline, associations for both TyGFI control levels and cumTyGFI were consistent with the main findings (Supplementary Table S5). When the wave-based time definition was applied instead of the midpoint approach, the estimates remained virtually unchanged (Supplementary Table S6). Collectively, these results confirm the stability and internal consistency of the study findings.

## Discussion

This study, based on a nationally representative cohort of middle-aged and older Chinese adults, systematically evaluated the longitudinal trajectories and cumulative burden of TyGFI in relation to hip fracture risk. The findings revealed that individuals with persistently elevated or progressively increasing TyGFI levels exhibited a markedly higher risk of hip fracture. Moreover, cumulative TyGFI was nonlinearly associated with fracture risk, with approximately 4.5 identified as a potential threshold. SHAP analysis further demonstrated that TyGFI contributed the most among all covariates, underscoring its pivotal role as a key predictor of hip fracture. Collectively, these results provide the first evidence from a long-term dynamic and cumulative exposure perspective linking the metabolic–frailty composite burden to skeletal fragility through a stable and independent association.

Insulin resistance and metabolic dysfunction may help explain the observed association between TyGFI and hip fracture risk. Chronic hyperglycemia and hyperlipidemia may contribute to persistent inflammation and oxidative stress, which can adversely affect bone remodeling and bone material properties. In this context, progressive accumulation of AGEs within long-lived bone matrix proteins, particularly type I collagen, may increase nonenzymatic collagen cross-linking, compromise bone toughness, and increase susceptibility to microdamage [15, 30]. AGE-related signaling disturbances may further impair osteoblast function and mineral deposition, collectively contributing to skeletal fragility even when BMD is relatively preserved [15, 30]. In parallel, dysregulated insulin signaling disrupts the bone–muscle axis, accelerates sarcopenia progression, and increases fall susceptibility, providing a plausible explanation for the “diabetic bone paradox,” whereby patients with diabetes exhibit normal bone mineral density but heightened fracture risk [17]. Compared with prior studies that relied mainly on single TyG measurements, the present study incorporated clustering-based TyGFI pattern classification and cumulative exposure assessment, offering a more comprehensive depiction of the dynamic and long-term effects of the metabolic–frailty burden [19, 31].

By integrating the triglyceride–glucose (TyG) index with the frailty index (FI), TyGFI is intended to reflect insulin resistance–related metabolic burden together with multisystem vulnerability captured by deficit accumulation. In the present cohort, participants classified into higher TyGFI control levels (moderate–increasing and high–increasing) experienced a higher hazard of incident hip fracture. In addition, greater cumTyGFI was associated with higher fracture risk, and adding TyGFI-related measures improved model discrimination and risk reclassification indices, indicating incremental prognostic information beyond conventional covariates. These findings are concordant with prior evidence linking TyG-related metabolic dysregulation to skeletal fragility. In a 6-year ambispective cohort of hospitalized postmenopausal women with T2DM and osteoporosis, higher TyG tertiles were associated with a greater risk of incident fragility fractures [18]. In a large general-population prospective cohort with repeated examinations, higher cumulative TyG exposure was also associated with an increased risk of onset fragility fractures after multivariable adjustment [32]. Notably, these prior studies focused on TyG or cumTyG and overall fragility fractures, whereas the present study evaluated an integrated metabolic–frailty construct (TyGFI/cumTyGFI) and hip fracture specifically, which may partly account for differences in the observed dose–response shape.

Frailty is a recognized determinant of falls, fractures, and adverse outcomes in older adults. A recent prospective UK Biobank study (median follow-up 13.6 years) reported that frailty was independently associated with increased risks of multiple fracture types, including hip fracture, and that inflammatory biomarkers (C-reactive protein, neutrophils, and platelets) explained a small but statistically significant proportion of the frailty–fracture association [33]. In addition, frailty has been consistently linked to worse outcomes after hip fracture, including substantially higher short-term postoperative all-cause and cause-specific mortality in nationwide registry data [34] and improved 30-day mortality risk stratification in surgical hip fracture cohorts using validated frailty scales [35]. Together with evidence that frailty predicts longer term mortality and rehospitalization after hip fracture surgery [36], these studies highlight the clinical relevance of frailty assessment. Building on this literature, the present study suggests that TyGFI may help identify community-dwelling individuals with concurrent metabolic vulnerability and physiological deficit burden prior to hip fracture occurrence, thereby supporting earlier risk stratification for prevention.

From a translational perspective, TyGFI is derived from routinely collected fasting glucose and triglycerides together with a reproducible deficit accumulation FI, which may enhance feasibility in epidemiologic and clinical risk stratification settings. Importantly, prior work suggests that frailty may complement conventional fracture prediction tools: in a 10-year longitudinal cohort of community-dwelling older women, incorporating a frailty index alongside FRAX yielded marginally improved discrimination for hip fracture compared with either measure alone and identified additional high-risk individuals among those otherwise categorized as lower risk [37]. In this context, TyGFI may represent another integrative approach to capture metabolic–frailty burden that is not reflected by bone density alone; however, its utility within established prediction frameworks warrants dedicated external validation and calibration. Mechanistically, diabetes-related skeletal fragility has been linked to the AGE–collagen axis, whereby AGE accumulation and nonenzymatic cross-linking impair mineralization and compromise bone material properties [38, 39]. These pathways, together with metabolic inflammation and musculoskeletal decline, provide biological plausibility for considering long-term metabolic–frailty burden when evaluating hip fracture risk.

This study has several notable strengths. First, it leveraged a nationally representative prospective cohort with long-term follow-up and repeated fasting glucose and triglyceride measurements, enabling evaluation of both cluster-based TyGFI control levels and cumulative exposure. Second, complementary analytic approaches were applied, and the associations remained consistent across prespecified subgroup, complete-case, alternative time-scale, and restriction analyses. Notably, findings were materially unchanged in analyses restricted to participants free of baseline diabetes and dyslipidemia, supporting that the observed associations were not driven solely by these baseline diagnoses.

Several limitations should be acknowledged. First, TyGFI is a recently proposed composite indicator, and published evidence remains limited; therefore, replication in diverse populations is warranted. Second, although FI is grounded in the widely used deficit accumulation framework, its operationalization may vary across cohorts depending on available variables, which may affect cross-study comparability. Third, hip fracture events were self-reported without radiographic confirmation, which may have introduced some degree of outcome misclassification. Fourth, TyGFI was assessed at only two time points and bone mineral density/turnover markers were unavailable, limiting the characterization of long-term metabolic–frailty dynamics and direct comparison with established skeletal predictors. Fifth, although physical activity was adjusted for, detailed nutritional measures (e.g., calcium/vitamin D intake) and specific weight-bearing or sedentary behavior metrics were not available in CHARLS, which may lead to residual confounding. Importantly, results remained consistent in sensitivity analyses restricted to participants free of both baseline diabetes and dyslipidemia, indicating that findings were not solely attributable to these baseline diagnoses. Future studies incorporating repeated measurements, imaging, and biomarker data are needed to further refine risk stratification and elucidate underlying mechanisms.

## Conclusion

In conclusion, this study provides novel evidence that long-term trajectories and cumulative exposure of metabolic–frailty burden are independently associated with hip fracture risk. These findings suggest that sustained metabolic–frailty imbalance may play a crucial role in the development of skeletal fragility. As an integrated indicator combining metabolic dysfunction and frailty status, TyGFI shows promising potential for incorporation into fracture risk assessment and warrants further validation in diverse populations.

## Supplementary Information

Below is the link to the electronic supplementary material.

ESM 1 (DOCX 1.37 MB)

## Data Availability

The data supporting the findings of this study are publicly available from the official CHARLS website (http://charls.pku.edu.cn). Access to these datasets is granted for research purposes upon request or registration, in compliance with the respective data use policies.
