# Supplementary material for: Long-term trajectories and cumulative exposure of the triglyceride–glucose–frailty index in relation to hip fracture risk: evidence from a large-scale population-based cohort
Source: Arch Osteoporos. 2026 May 8;21(1):80. doi: 10.1007/s11657-026-01683-z (PMC13156187; doi:10.1007/s11657-026-01683-z)
Supplement: Supplementary file 1 — (DOCX 1.37 MB) [file 11657_2026_1683_MOESM1_ESM.docx]

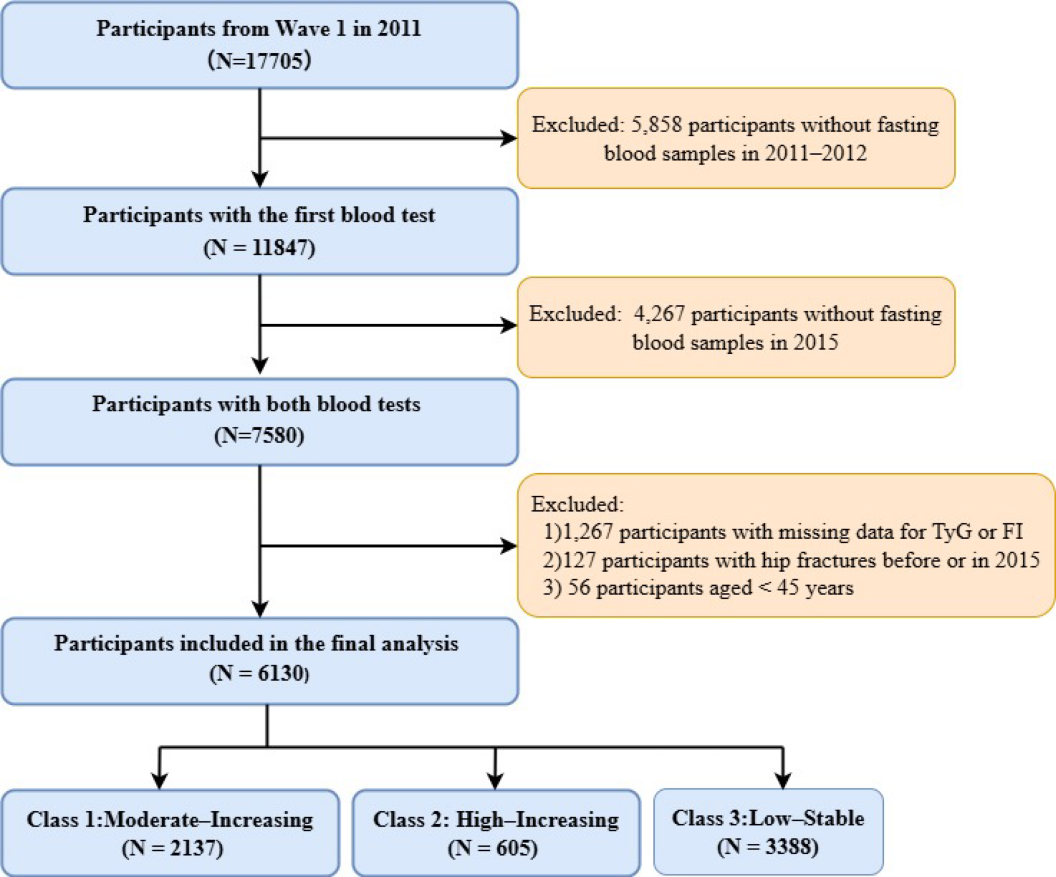


**SI1** Flow diagram of participant inclusion and exclusion in the CHARLS cohort for analyses of TyGFI trajectories and cumulative exposure in relation to incident hip fracture

**
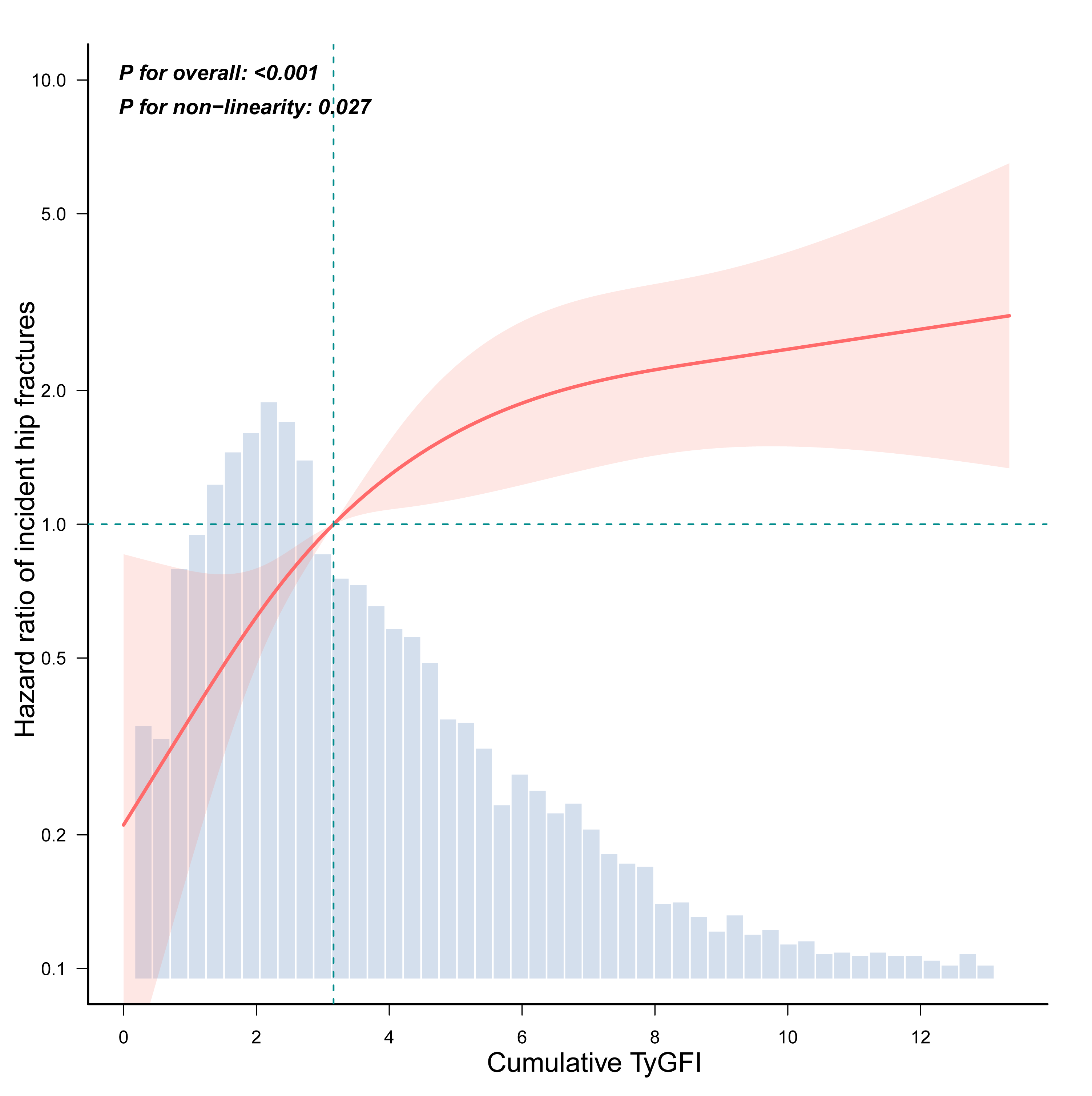
**

**SI2** Restricted cubic spline (RCS) analysis illustrating the non-linear association between cumulative TyGFI and the risk of incident hip fracture, based on the fully adjusted Cox proportional hazards model (Model 3). The red line represents the estimated hazard ratio (HR), and the shaded area indicates the 95% confidence interval. The histogram shows the distribution of cumulative TyGFI values in the study population


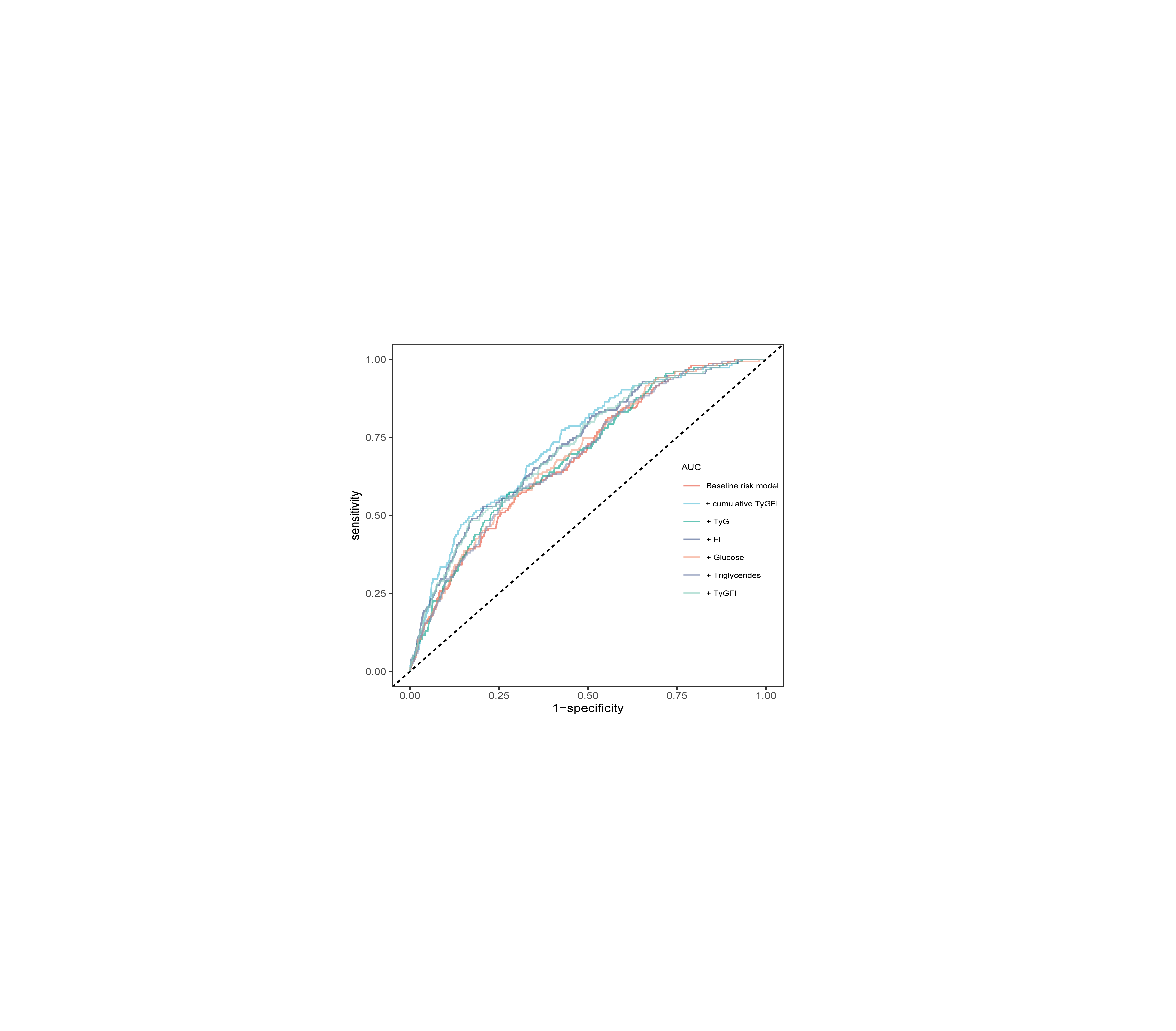


**SI3** Receiver operating characteristic (ROC) curves assessing the incremental predictive value of metabolic and frailty indicators beyond the baseline risk model.The baseline model (red line) included age, sex, education level, marital status, residence, smoking, drinking, comorbidity, and arthritis (Model 3).Extended models were created by adding one variable at a time: cumTyGFI, TyGFI, FI, TyG, fasting glucose, or triglycerides.The model incorporating cumTyGFI showed the greatest improvement in discrimination (AUC = 0.734)

**
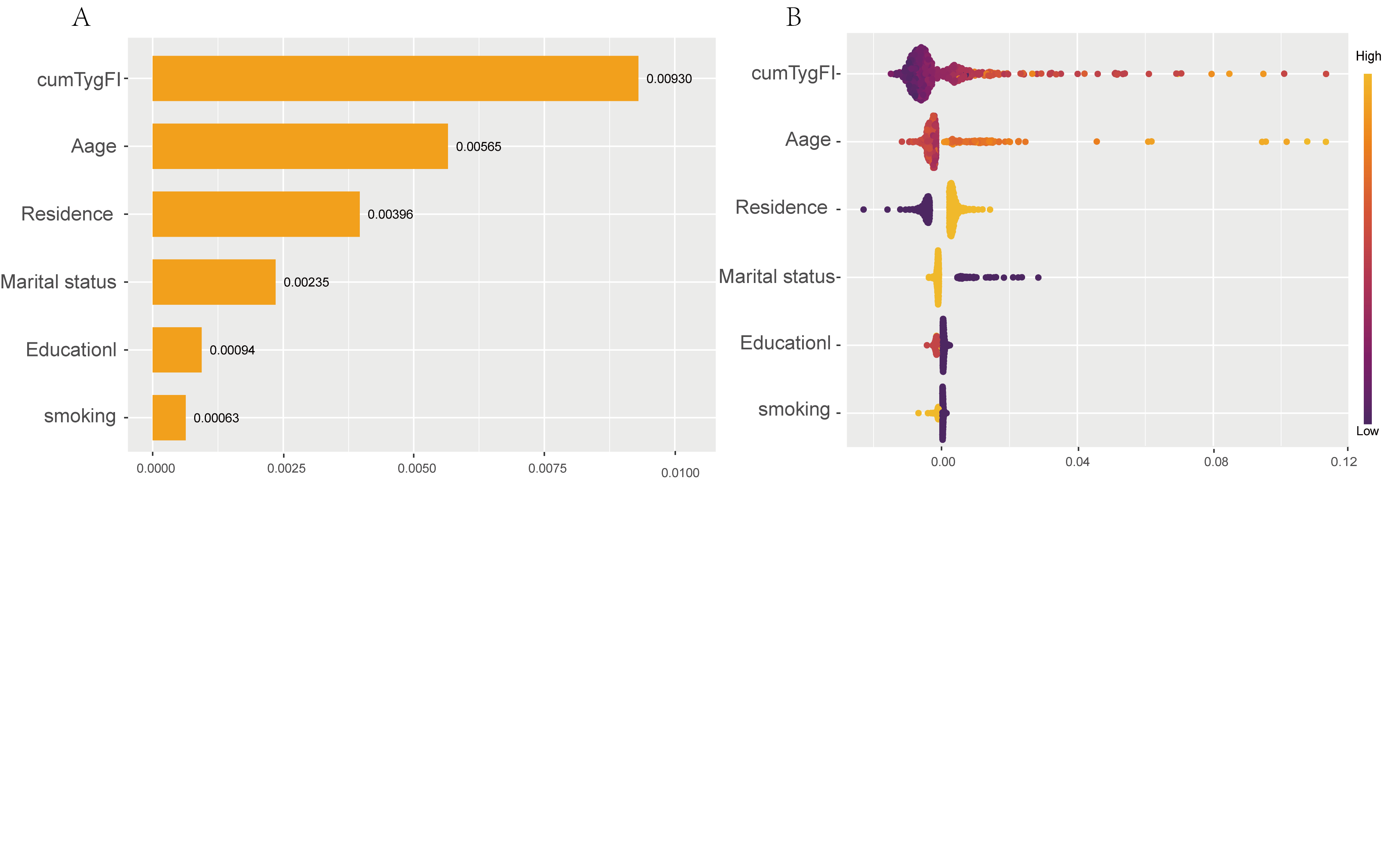
**

**SI4** SHAP (SHapley Additive exPlanations) analysis of model feature importance for predicting hip fracture. (A) Mean absolute SHAP values showing each feature’s overall contribution; cumTyGFI was the top predictor. (B) SHAP summary plot illustrating the direction and magnitude of variable contributions. Each dot represents one individual; color denotes feature value (yellow = high, purple = low)

****Supplementary** table S1** Deficits included in the Frailty Index (FI) in CHARLS and their cut-off values.

| **No.** | **Description of the item** | **Cutoff value / Scoring criteria** |
| --- | --- | --- |
| 1 | Hypertension diagnosed by a physician | Yes = 1; No = 0 |
| 2 | Diabetes diagnosed by a physician | Yes = 1; No = 0 |
| 3 | Heart disease (including angina, coronary heart disease, or heart failure) | Yes = 1; No = 0 |
| 4 | Stroke diagnosed by a physician | Yes = 1; No = 0 |
| 5 | Any type of cancer diagnosis | Yes = 1; No = 0 |
| 6 | Arthritis | Yes = 1; No = 0 |
| 7 | Chronic lung disease | Yes = 1; No = 0 |
| 8 | Asthma | Yes = 1; No = 0 |
| 9 | Emotional, nervous, or psychiatric disorders | Yes = 1; No = 0 |
| 10 | Memory-related disease (e.g., Alzheimer’s disease, dementia) | Yes = 1; No = 0 |
| 11 | Self-reported vision problems | Yes = 1; No = 0 |
| 12 | Self-reported hearing problems | Yes = 1; No = 0 |
| 13 | Self-rated general health | Poor or fair = 1; good, very good, or excellent = 0 |
| 14 | Difficulty dressing | Yes = 1; No = 0 |
| 15 | Difficulty bathing or showering | Yes = 1; No = 0 |
| 16 | Difficulty eating | Yes = 1; No = 0 |
| 17 | Difficulty getting in or out of bed | Yes = 1; No = 0 |
| 18 | Difficulty using the toilet | Yes = 1; No = 0 |
| 19 | Difficulty managing money | Yes = 1; No = 0 |
| 20 | Difficulty taking medications | Yes = 1; No = 0 |
| 21 | Difficulty shopping for groceries | Yes = 1; No = 0 |
| 22 | Difficulty preparing meals | Yes = 1; No = 0 |
| 23 | Difficulty doing housework | Yes = 1; No = 0 |
| 24 | Difficulty walking 100 meters | Yes = 1; No = 0 |
| 25 | Difficulty rising from a chair after sitting for a long time | Yes = 1; No = 0 |
| 26 | Difficulty climbing several flights of stairs without resting | Yes = 1; No = 0 |
| 27 | Difficulty lifting or carrying weights > 10 pounds (≈ 5 kg) | Yes = 1; No = 0 |
| 28 | Difficulty picking up a small object from a table | Yes = 1; No = 0 |
| 29 | Difficulty stooping, kneeling, or crouching | Yes = 1; No = 0 |
| 30 | Difficulty reaching arms above shoulder level | Yes = 1; No = 0 |
| 31 | Depressive symptoms (CESD-10 score) | CESD-10 > 10 = 1; ≤ 10 = 0 |
| 32 | Cognitive function score (memory + orientation tests)/4 | Continuous (0–1) |

Heart disease refers to physician-diagnosed angina, coronary heart disease, congestive heart failure, or other heart problems. Memory-related disease includes Alzheimer’s disease, dementia, organic brain degeneration, or other severe memory disorders. Depressive symptoms were assessed by the 10-item Center for Epidemiologic Studies Depression Scale (CESD-10), ranging from 0–30, with higher scores indicating greater symptom burden. Cognitive function was evaluated from immediate and delayed word recall (0–10 points) and temporal orientation (day, month, date, year; 0–4 points), standardized to a 0–1 scale. FI was calculated as a continuous proportion score (deficits/total). No threshold-based categorization was used in the main analyses. For interpretation, prior deficit-accumulation studies often report an empirical frailty threshold around FI ≥ 0.25.

****Supplementary** table S2** Baseline characteristics of participants overall and by TyGFI trajectory class

| **Variables** | Overall  (n = 6130) | Low–Stable  (n = 3388) | Moderate–Increasing  (n = 2137) | | High–Increasing  (n = 605) | *P* value |
| --- | --- | --- | --- | --- | --- | --- |
| **Age(year)** | 58.0 ± 8.3 | 56.5 ± 8.0 | 59.2 ± 8.3 | 62.0 ± 8.2 | | < 0.001 |
| **Sex,n (%)** | | | | | | < 0.001 |
| Male | 2726 (44.5) | 1773 (52.3) | 776 (36.3) | 177 (29.3) | |  |
| Female | 3404 (55.5) | 1615 (47.7) | 1361 (63.7) | 428 (70.7) | |  |
| **Education level,n (%)** | | | | | | < 0.001 |
| Primary school or below | 4284 (69.9) | 2073 (61.2) | 1682 (78.7) | 529 (87.4) | |  |
| High school | 1674 (27.3) | 1184 (34.9) | 417 (19.5) | 73 (12.1) | |  |
| College or above | 172 ( 2.8) | 131 (3.9) | 38 (1.8) | 3 (0.5) | |  |
| **Married, n (%)** | | | | | | < 0.001 |
| No | 855 (13.9) | 381 (11.2) | 366 (17.1) | 108 (17.9) | |  |
| Yes | 5275 (86.1) | 3007 (88.8) | 1771 (82.9) | 497 (82.1) | |  |
| **Residence ,n (%)** | | | | | | < 0.001 |
| Urban | 2030 (33.1) | 1235 (36.5) | 633 (29.6) | 162 (26.8) | |  |
| Rural | 4100 (66.9) | 2153 (63.5) | 1504 (70.4) | 443 (73.2) | |  |
| **Alcohol consumption,n (%)** | | | | | | < 0.001 |
| Never | 4372 (71.3) | 2219 (65.5) | 1645 (77) | 508 (84) | |  |
| Drink but less than once a month | 505 ( 8.2) | 311 (9.2) | 156 (7.3) | 38 (6.3) | |  |
| Drink more than once a month | 1253 (20.4) | 858 (25.3) | 336 (15.7) | 59 (9.8) | |  |
| **Smoking status,n (%)** | | | | | | < 0.001 |
| No | 4337 (70.8) | 2241 (66.1) | 1609 (75.3) | 487 (80.5) | |  |
| Yes | 1793 (29.2) | 1147 (33.9) | 528 (24.7) | 118 (19.5) | |  |
| **Physical activity, n (%)** | | | | | | 0.002 |
| Light | 4377 (71.4) | 2382 (70.3) | 1527 (71.5) | 468 (77.4) | |  |
| Moderate-to-vigorous | 1753 (28.6) | 1006 (29.7) | 610 (28.5) | 137 (22.6) | |  |
| **Triglycerides(mg/dL)** | 106.2 (75.2, 156.6) | 99.1 (71.7, 146.9) | 111.5 (78.8, 162.8) | 127.4 (92.9, 185.0) | | < 0.001 |
| **Glucose(mg/dL)** | 110.0 ± 35.4 | 107.3 ± 30.2 | 111.9 ± 39.8 | 118.4 ± 43.3 | | < 0.001 |
| **TyGFI_2012_** | 0.9 (0.4, 1.6) | 0.5 (0.3, 0.8) | 1.5 (1.1, 1.9) | 3.0 (2.4, 3.7) | | < 0.001 |
| **TyGFI_2015_** | 1.2 (0.7, 2.0) | 0.7 (0.4, 1.0) | 1.9 (1.5, 2.4) | 3.6 (2.8, 4.5) | | < 0.001 |
| **Frailty index** | 0.1 (0.0, 0.2) | 0.1 (0.0, 0.1) | 0.2 (0.1, 0.2) | 0.3 (0.3, 0.4) | | < 0.001 |
| **Hypertension,n (%)** | | | | | | < 0.001 |
| No | 4474 (73.4) | 2849 (84.6) | 1337 (62.9) | 288 (48) | |  |
| Yes | 1619 (26.6) | 519 (15.4) | 788 (37.1) | 312 (52) | |  |
| **Diabetes,n (%)** | | | | | | < 0.001 |
| No | 5697 (93.9) | 3263 (97.1) | 1922 (91.2) | 512 (85.3) | |  |
| Yes | 370 ( 6.1) | 97 (2.9) | 185 (8.8) | 88 (14.7) | |  |
| **Dyslipidemia,n (%)** | | | | | | < 0.001 |
| No | 5353 (89.2) | 3090 (92.8) | 1791 (85.9) | 472 (80.4) | |  |
| Yes | 650 (10.8) | 240 (7.2) | 295 (14.1) | 115 (19.6) | |  |
| **Arthritis,n (%)** | | | | | | < 0.001 |
| No | 3193 (52.8) | 2354 (70.4) | 718 (34.1) | 121 (20.2) | |  |
| Yes | 2853 (47.2) | 989 (29.6) | 1386 (65.9) | 478 (79.8) | |  |
| **Comorbidity^a^,n (%)** | | | | | | < 0.001 |
| 0 | 1896 (30.9) | 1623 (47.9) | 243 (11.4) | 30 (5) | |  |
| 1 | 2313 (37.7) | 1323 (39) | 842 (39.4) | 148 (24.5) | |  |
| 2 | 1921 (31.3) | 442 (13) | 1052 (49.2) | 427 (70.6) | |  |

^a^Comorbidity was categorized according to the number of nine chronic conditions (hypertension, diabetes, dyslipidemia, chronic kidney disease, heart disease, stroke, chronic lung disease, arthritis, and cancer), defined as 0, 1, or ≥2. TyGFI, TyGFrailty index

****Supplementary** table S3** Subgroup analyses for the association between TyGFI trajectory classes and incident hip fracture (Model 3).

| **Variable** | **class3** | **class1** | **class2** | ***P* for interaction** |
| --- | --- | --- | --- | --- |
| **Age(year)** |  |  |  | 0.711 |
| **<60** | 1(Ref) | 1.78 (0.97~3.28) | 2.57 (1.11~5.92) |  |
| **>=60** | 1(Ref) | 2.03 (1.13~3.64) | 4.83 (2.49~9.36) |  |
| **Sex** |  |  |  | 0.802 |
| **Male** | 1(Ref) | 2.04 (1.09~3.79) | 3.32 (1.45~7.6) |  |
| **Female** | 1(Ref) | 1.92 (1.07~3.44) | 4.2 (2.14~8.26) |  |
| **Education** |  |  |  | 0.702 |
| **Primary school or below** |  | 2.22 (1.4~3.54) | 4.22 (2.42~7.35) |  |
| **High school or above** |  | 1.11 (0.36~3.4) | 2.96 (0.62~14.25) |  |
| **Residence** |  |  |  | 0.886 |
| **Urban** | 1(Ref) | 2.49 (0.94~6.6) | 5.55 (1.65~18.68) |  |
| **Rural** | 1(Ref) | 1.9 (1.19~3.04) | 3.7 (2.09~6.55) |  |
| **BMI** |  |  |  | 0.533 |
| **Underweight** |  | 3.28 (0.34~31.94) | 8.52 (0.54~135.15) |  |
| **Normal** | 1(Ref) | 1.61 (0.93~2.77) | 2.81 (1.39~5.68) |  |
| **Overweight** | 1(Ref) | 2.23 (0.76~6.53) | 6.67 (2.01~22.16) |  |
| **Drinking status** |  |  |  | 0.693 |
| **Never** | 1(Ref) | 2.36 (1.39~3.99) | 5.12 (2.78~9.42) |  |
| **Drink but less than once a month** | 1(Ref) | 3.19 (0.49~20.8) | 2.13 (0.13~36.13) |  |
| **Drink more than once a month** | 1(Ref) | 1.31 (0.59~2.89) | 1.74 (0.46~6.5) |  |
| **Smoking status** |  |  |  | 0.268 |
| **No** | 1(Ref) | 2.21 (1.33~3.67) | 3.83 (2.05~7.14) |  |
| **Yes** | 1(Ref) | 1.54 (0.71~3.35) | 4.04 (1.63~10.04) |  |
| **Arthritis** |  |  |  | 0.158 |
| **No** | 1(Ref) | 2.9 (1.62~5.18) | 4.37 (1.78~10.71) |  |
| **Yes** | 1(Ref) | 1.29 (0.73~2.3) | 2.85 (1.5~5.4) |  |

Analyses were based on model 3, with the stratification variable itself not included in the adjustment.

****Supplementary** table S4** Associations of TyGFI control levels and cumulative TyGFI with risk of hip fractures in a complete-case sample (N = 5575).

| **Variables** | **Crude Model** | |  | **Model 1** | |  | **Model 2** | |  | **Model 3** | |
| --- | --- | --- | --- | --- | --- | --- | --- | --- | --- | --- | --- |
|  | **HR (95% CI)** | *P*-Value |  | **HR (95% CI)** | *P*-Value |  | **HR (95% CI)** | *P*-Value |  | **HR (95% CI)** | *P*-Value |
| **TyGFI control levels** | |  |  |  |  |  |  |  |  |  |  |
| **Class 3** | 1(Ref) |  |  | 1(Ref) |  |  | 1(Ref) |  |  | 1(Ref) |  |
| **Class 1** | 2.2 (1.5~3.22) | <0.001 |  | 1.78 (1.2~2.65) | 0.004 |  | 1.82 (1.23~2.7) | 0.003 |  | 1.9 (1.22~2.94) | 0.004 |
| **Class 2** | 4.56 (2.94~7.07) | <0.001 |  | 3.33 (2.1~5.28) | <0.001 |  | 3.43 (2.16~5.46) | <0.001 |  | 3.71 (2.17~6.34) | <0.001 |
|  |  |  |  |  |  |  |  |  |  |  |  |
| **cumTyGFI Continuous** | 1.18 (1.14~1.23) | <0.001 |  | 1.16 (1.1~1.21) | <0.001 |  | 1.16 (1.11~1.22) | <0.001 |  | 1.18 (1.12~1.24) | <0.001 |
| **cumTyGFI Quartile** | |  |  |  |  |  |  |  |  |  |  |
| **Q1** | 1(Ref) |  |  | 1(Ref) |  |  | 1(Ref) |  |  | 1(Ref) |  |
| **Q2** | 1.84 (0.94~3.61) | 0.077 |  | 1.6 (0.81~3.14) | 0.175 |  | 1.62 (0.82~3.18) | 0.165 |  | 1.8 (0.9~3.62) | 0.097 |
| **Q3** | 2.84 (1.51~5.35) | 0.001 |  | 2.23 (1.17~4.22) | 0.014 |  | 2.29 (1.21~4.36) | 0.011 |  | 2.65 (1.32~5.29) | 0.006 |
| **Q4** | 5.34 (2.95~9.66) | <0.001 |  | 3.8 (2.06~7.01) | <0.001 |  | 3.94 (2.13~7.28) | <0.001 |  | 4.89 (2.43~9.83) | <0.001 |

Model 1 was adjusted for age, sex, education level, marital status and residence; Model 2 was further adjusted for smoking status, alcohol consumption and physical activity; Model 3 was further adjusted for comorbidity (0/1/≥2) and arthritis; HR, Hazard ratio; CI, Confidence interval; Ref, Reference; cumTyGFI, cumulative triglyceride-glucose and frailty index.

Supplementary Table S5. Associations of TyGFI trajectory groups and cumulative TyGFI exposure (cumTyGFI) with incident hip fracture among participants free of both diabetes and dyslipidemia at baseline.

| **Variables** | **Crude Model** | |  | **Model 1** | |  | **Model 2** | |  | **Model 3** | |
| --- | --- | --- | --- | --- | --- | --- | --- | --- | --- | --- | --- |
|  | **HR (95% CI)** | P **Value** |  | **HR (95% CI)** | P **Value** |  | **HR (95% CI)** | P **Value** |  | **HR (95% CI)** | P **Value** |
| **TyGFI control levels** | |  |  |  |  |  |  |  |  |  |  |
| **Class 3(Low–Stable)** | 1(Ref) |  |  | 1(Ref) |  |  | 1(Ref) |  |  | 1(Ref) |  |
| **Class 1(Moderate–Increasing)** | 2.46 (1.68~3.6) | <0.001 |  | 1.96 (1.32~2.9) | 0.001 |  | 1.99 (1.34~2.95) | 0.001 |  | 2.05 (1.33~3.17) | 0.001 |
| **Class 2(High–Increasing)** | 5.32 (3.43~8.24) | <0.001 |  | 3.69 (2.32~5.86) | <0.001 |  | 3.82 (2.4~6.09) | <0.001 |  | 4.1 (2.41~6.98) | <0.001 |
|  |  |  |  |  |  |  |  |  |  |  |  |
| **cumTyGFI Continuous** | 1.2 (1.15~1.25) | <0.001 |  | 1.16 (1.11~1.22) | <0.001 |  | 1.17 (1.12~1.23) | <0.001 |  | 1.19 (1.12~1.25) | <0.001 |
| **cumTyGFI Quartile** | |  |  |  |  |  |  |  |  |  |  |
| **Q1** | 1(Ref) |  |  | 1(Ref) |  |  | 1(Ref) |  |  | 1(Ref) |  |
| **Q2** | 2.05 (1.02~4.09) | 0.043 |  | 1.78 (0.89~3.56) | 0.105 |  | 1.79 (0.89~3.58) | 0.102 |  | 1.97 (0.96~4.01) | 0.063 |
| **Q3** | 3.67 (1.93~6.97) | <0.001 |  | 2.81 (1.46~5.39) | 0.002 |  | 2.88 (1.5~5.53) | 0.002 |  | 3.26 (1.62~6.57) | 0.001 |
| **Q4** | 6.45 (3.49~11.92) | <0.001 |  | 4.38 (2.32~8.25) | <0.001 |  | 4.53 (2.4~8.56) | <0.001 |  | 5.5 (2.7~11.2) | <0.001 |

Model 1 was adjusted for age, sex, education level, marital status and residence; Model 2 was further adjusted for smoking status, alcohol consumption and physical activity; Model 3 was further adjusted for comorbidity (0/1/≥2) and arthritis; HR, Hazard ratio; CI, Confidence interval; Ref, Reference; cumTyGFI, cumulative triglyceride-glucose and frailty index.

****Supplementary** table S6** Associations of TyGFI Trajectory Classes and Cumulative TyGFI (cumTyGFI) with the Risk of Incident Hip Fracture Using a Wave-Based Time Definition.

| **Variables** | **Crude Model** | |  | **Model 1** | |  | **Model 2** | |  | **Model 3** | |
| --- | --- | --- | --- | --- | --- | --- | --- | --- | --- | --- | --- |
|  | **HR (95% CI)** | ***P* Value** |  | **HR (95% CI)** | ***P* Value** |  | **HR (95% CI)** | ***P* Value** |  | **HR (95% CI)** | ***P* Value** |
| **TyGFI control levels** | |  |  |  |  |  |  |  |  |  |  |
| **Class 3(Low–Stable)** | 1(Ref) |  |  | 1(Ref) |  |  | 1(Ref) |  |  | 1(Ref) |  |
| **Class 1(Moderate–Increasing)** | 2.34 (1.61~3.39) | <0.001 |  | 1.92 (1.31~2.81) | 0.001 |  | 1.95 (1.33~2.86) | 0.001 |  | 1.97 (1.29~3.01) | 0.002 |
| **Class 2(High–Increasing)** | 4.95 (3.25~7.55) | <0.001 |  | 3.59 (2.3~5.6) | <0.001 |  | 3.68 (2.35~5.75) | <0.001 |  | 3.77 (2.25~6.31) | <0.001 |
|  |  |  |  |  |  |  |  |  |  |  |  |
| **cumTyGFI Continuous** | 1.19 (1.15~1.24) | <0.001 |  | 1.16 (1.11~1.22) | <0.001 |  | 1.17 (1.12~1.22) | <0.001 |  | 1.18 (1.12~1.24) | <0.001 |
| **cumTyGFI Quartile** | |  |  |  |  |  |  |  |  |  |  |
| **Q1** | 1(Ref) |  |  | 1(Ref) |  |  | 1(Ref) |  |  | 1(Ref) |  |
| **Q2** | 2.14 (1.08~4.25) | 0.026 |  | 1.89 (0.95~3.75) | 0.065 |  | 1.9 (0.96~3.77) | 0.067 |  | 2.1 (1.04~4.24) | 0.039 |
| **Q3** | 3.49 (1.84~6.64) | <0.001 |  | 2.77 (1.44~5.3) | 0.002 |  | 2.83 (1.48~5.42) | 0.002 |  | 3.18 (1.58~6.41) | 0.001 |
| **Q4** | 6.28 (3.42~11.56) | <0.001 |  | 4.47 (2.39~8.37) | <0.001 |  | 4.6 (2.45~8.63) | <0.001 |  | 5.45 (2.68~11.06) | <0.001 |

Model 1 was adjusted for age, sex, education level, marital status and residence; Model 2 was further adjusted for smoking status, alcohol consumption and physical activity; Model 3 was further adjusted for comorbidity (0/1/≥2) and arthritis; HR, Hazard ratio; CI, Confidence interval; Ref, Reference; cumTyGFI, cumulative triglyceride-glucose and frailty index.
